# Association of Lipid Peroxidation Product 4-Hydroxynonenal with Post-Traumatic Stress Disorder †

**DOI:** 10.3390/biom11091365

**Published:** 2021-09-15

**Authors:** Matea Nikolac Perković, Lidija Milković, Suzana Uzun, Ninoslav Mimica, Nela Pivac, Georg Waeg, Neven Žarković

**Affiliations:** 1 Laboratory of Molecular Neuropsychiatry, Division of Molecular Medicine, Rudjer Boskovic Institute, Bijenička 54, 10000 Zagreb, Croatia; mnikolac@irb.hr (M.N.P.); npivac@irb.hr (N.P.); 2 Laboratory for Oxidative Stress (LabOS), Division of Molecular Medicine, Rudjer Boskovic Institute, Bijenička 54, 10000 Zagreb, Croatia; lidija.milkovic@irb.hr; 3 Department for Biological Psychiatry and Psychogeriatrics, University Psychiatric Hospital Vrapče, 10090 Zagreb, Croatia; suzana.uzun@gmail.com (S.U.); nino.mimica@gmail.com (N.M.); 4 School of Medicine, University of Zagreb, 10000 Zagreb, Croatia; 5 Institute of Molecular Biosciences, Karl Franzens University of Graz, Heinrichstraße 31/II, 8010 Graz, Austria; georg.waeg@uni-graz.at

**Keywords:** posttraumatic stress disorder (PTSD), 4-hydroxynonenal (4-HNE), oxidative stress, lipid peroxidation, ELISA, biomarker, human plasma samples

## Abstract

Repeated activation of the hypothalamic-pituitary-adrenal axis system, sleep disturbances, and other symptoms related to posttraumatic stress disorder (PTSD) elevate reactive oxygen species, increase inflammation, and accelerate cellular aging, leading to neuroprogression and cognitive decline. However, there is no information about possible involvement of 4-hydroxynonenal (4-HNE), the product of lipid peroxidation associated with stress-associated diseases, in the complex etiology of PTSD. Therefore, the aim of this study was to compare the plasma levels of 4-HNE between war veterans with PTSD (*n* = 62) and age-, sex- and ethnicity- matched healthy control subjects (*n* = 58) in order to evaluate the potential of HNE-modified proteins as blood-based biomarker of PTSD. The genuine 4-HNE-Enzyme-Linked Immunosorbent Assay (HNE-ELISA), based on monoclonal antibody specific for HNE-histidine (HNE-His) adducts, was used to determine plasma HNE-protein conjugates. Our results revealed significantly elevated levels of 4-HNE in patients with PTSD. Moreover, the accumulation of plasma 4-HNE seems to increase with aging but in a negative correlation with BMI, showing specific pattern of change for individuals diagnosed with PTSD. These findings suggest that oxidative stress and altered lipid metabolism reflected by increase of 4-HNE might be associated with PTSD. If confirmed with further studies, elevated 4-HNE plasma levels might serve as a potential biomarker of PTSD.

## 1. Introduction

Posttraumatic stress disorder (PTSD) is a trauma and stress-related disorder [1] that occurs in a portion of individuals exposed to a traumatic experience or traumatic events. The prevalence of PTSD differs between different countries and between civilian and military population. However, the lifetime prevalence of PTSD in the general population is estimated at about 8% [2]. The prevalence of combat-related PTSD in the United States is approximately 11–20% [3], while the prevalence of PTSD in Croatian war veterans is estimated between 14–40% [4]. PTSD is a serious and often disabling condition comorbid with different psychiatric and somatic comorbidities, such as cardiovascular and pulmonary diseases, metabolic syndrome, increased inflammation, and autoimmune disorders [5]. It was suggested to represent a chronic state of sustained stress [6]. Repeated activation of the hypothalamic-pituitary-adrenal (HPA) axis system, sleep disturbances, and other PTSD-related symptoms elevate reactive oxygen species (ROS), increase inflammation, and also accelerate cellular aging, resulting in neuroprogression and cognitive decline [7]. PTSD was associated with accelerated aging due to disrupted neural integrity and cognitive disturbances [8].

Oxidative stress is a molecular mechanism implicated in different diseases and aging itself, co-occurring and intertwining with inflammation. Prolonged stressful events in childhood and adolescence have been associated with the persistent state of oxidative stress in the brain tissue and with the increased risk of developing certain psychiatric disorders [9]. Evidence from different studies supports the involvement of oxidative stress in PTSD pathophysiology. In Croatian war veterans, combat-related PTSD was associated with increased levels of glycerophospholipids, phosphatidylethanolamine (PE; 18:1/0:0), and phosphatidylcholine (PC; 18:1/0:0), which are associated with inflammation, mitochondrial dysfunction, membrane breakdown, oxidative stress, and neurotoxicity [10]. Lower concentrations of superoxide dismutase (SOD) and glutathione peroxidase (GPx), detected in erythrocytes from subjects with PTSD, suggest an impaired response to oxidative stress [11]. However, no significant differences in serum malondialdehyde (MDA) levels between war veterans with and without PTSD indicate a possible compensatory mechanism as an adaptive response to stress [11]. The role of oxidative stress in PTSD was also supported by evidence of elevated lipid peroxidation levels in serum samples from earthquake survivors that developed PTSD, along with the decreased activity of antioxidant enzymes [12]. Measurement of glutathione S-transferase mu1 levels, an enzyme that plays a key role in the detoxification of oxidative stress products, has been proposed as a potential marker to predict the development of PTSD in U.S. Marines [13,14]. However, similar urinary concentrations of 8-hydroxy-2′-deoxyguanosine, serum thromboxane B2, and serum urates were detected in war veterans with PTSD, with only slightly reduced concentration of protein carbonyls, suggesting that these selected markers of oxidative stress are not associated with PTSD [15].

The reactive aldehyde 4-hydroxynonenal (4-HNE) and its protein adducts are major bioactive products of the polyunsaturated fatty acids (PUFAs) peroxidation with biomarker potential in age- and stress-related diseases, especially due to its high affinity for proteins, this generating relatively stable adducts with histidine residues. The 4-HNE is a pleiotropic lipid peroxidation marker [16,17] that participates in various (patho)physiological processes due to its ability to interfere with signal transduction and activities of major biomolecules inducing inflammation [18,19] and acting as a second messenger of free radicals. So far, the role of 4-HNE has been extensively studied in neurodegenerative diseases, such as Alzheimer’s disease [20] and Parkinson’s disease, revealing its appearance within the brain blood vessels and neurons, even before the onset of clinical symptoms [21,22]. Moreover, 4-HNE is known to alter permeability of the blood-brain barrier during oxidative stress, thus penetrating into the brain from the blood vessels [23,24]. Under such circumstances, it could trigger the vitious circle of lipid peroxidation within the brain, which may be important for the pathology of the neurodegenerative diseases, trauma, and shock as well as for inflammatory processes and even brain tumors [16,23,25,26].

However, we are lacking information about possible involvement of 4-HNE in the complex etiology of PTSD. Therefore, the aim of this study was to compare the plasma levels of 4-HNE between war veterans with PTSD and age-, sex-, and ethnicity-matched healthy control subjects in order to evaluate the potential of HNE-modified proteins as blood-based biomarkers of PTSD.

## 2. Materials and Methods

### 2.1. Participants

The study included 120 age-matched and unrelated male Caucasian subjects of Croatian origin, 62 individuals with combat-related PTSD, and 58 healthy control subjects. Subjects were enrolled in the period between autumn of 2015 and spring of 2017. Individuals with combat-related PTSD were diagnosed using the Structured Clinical Interview (SCID) based on the Diagnostic and Statistical Manual of Mental Disorders (DSM-5) criteria [1]. At the time of sampling, subjects with PTSD were not receiving any psychiatric medication for at least 30 days. Individuals with PTSD were 38–75 years old and experienced war-related trauma during the Homeland War in Croatia (1991–1995). Healthy control subjects were 38–76 years old and were evaluated with the same diagnostic instruments as PTSD subjects. Enrollment of both groups of subjects followed the same inclusion/exclusion criteria. Exclusion criteria for all subjects were chronic drug abuse, alcohol dependence, diagnosis of depression, schizophrenia, bipolar disorder, adult attention deficit hyperactivity disorder, Alzheimer’s disease, current or recent (previous 3 months) alcohol abuse, and/or the use of lipid lowering agents and antihypertensive and antidiabetic medication. All participants were additionally evaluated according to the International Classification of Diseases (ICD-10) to exclude potential somatic diseases, such as fibrosis, sclerosis, cirrhosis, and malignant liver disease (alcoholic liver cirrhosis (K70.3), alcoholic liver fibrosis and sclerosis (K70.2), and hepatocellular carcinoma (C22.0)). The aims and procedures of the study were explained in detail to all participants. Written informed consent was obtained from all subjects and care was taken to ensure that all procedures contributing to this work agree with the ethical standards of the relevant institutional and national human research ethics committees. All procedures were approved by the Ethics Committee of the University Psychiatric Hospital Vrapce, Zagreb, Croatia, and they were consistent with the ethical norms and standards laid down in the Declaration of Helsinki.

### 2.2. Blood Sampling

Blood samples were collected between 7:30 and 8:00 a.m. after overnight fasting, using BD Vacutainer™ glass blood-collection tubes (Becton, Dickinson and Company, Franklin Lakes, NJ, USA) with acid citrate dextrose (ACD). Immediately after blood sampling, platelet-poor plasma was separated by series of centrifugation and stored at −80 °C.

### 2.3. Anthropometric and Biochemical Measurements

Biochemical and anthropometric characteristics were determined at the Laboratory of the University Psychiatric Hospital Vrapce. Height was measured to the nearest 0.5 cm. Body weight was measured with a digital scale to the nearest 0.1 kg. BMI was calculated as weight (kg) divided by height (m^2^).

Lipid levels were determined using commercial tests and Siemens Dimension Xpand analyzer. Total cholesterol (normal values < 5 mmol/L) was determined with cholesterol oxidase-phenol aminophenazone method, while triglycerides-TG (normal values < 1.7 mmol/L), low-density lipoprotein-LDL (normal values < 3 mmol/L), and high-density lipoprotein-HDL (normal values > 1.2 mmol/L) were determined using the enzymatic colorimetric assay. Fasting glucose level (normal values 4.4–6.4 mmol/L) was determined using hexokinase (Glucose System Reagent 800, Olympus AU640, Olympus America Inc., Center Valley, PA, USA) and was read on a Dimension Xpand Plus Integrated Chemistry System.

### 2.4. 4-HNE-ELISA

The level of 4-HNE protein conjugates was determined by an in-house protocol [27,28]. Briefly, the concentration of plasma samples, quantified by the Bradford method [29] and standards ranging from 0 to 250 pmol of 4-HNE-bovine serum albumin (BSA) conjugates/mg of protein, was adjusted to 10 mg/mL. Each standard/diluted plasma sample (10 μL) was added into 100 μL of 0.05 M carbonate binding buffer (pH 9.6; 0.015 M sodium carbonate, 0.035 M sodium bicarbonate) per well of an ELISA plate (Nunc Immuno Maxisorp, Thermo Scientific, Nunc A/S, 4000 Roskilde, Denmark) in triplicate. Thus, the prepared plate was incubated for 5h at 4 °C, washed once with phosphate-buffered saline (PBS; 200 μL), blocked with a blocking solution (5% fat-free dry milk in carbonate binding buffer) for 3h at room temperature (RT), and washed once with washing buffer (0.1% Tween 20 in PBS). Primary antibody (a generous gift from Dr. Waeg, [30]) (1:100) in 1% BSA in PBS was incubated overnight (ON) at 4 °C. To eliminate sample background values, one well of each sample was incubated with 1% BSA in PBS (without primary antibody). The next day, the plate was incubated with peroxidase blocking solution (3% H_2_O_2_ in blocking solution) for 30 min at RT, goat anti-mouse secondary antibody solution in 1% BSA in PBS (1:100; Dako) for 1h at RT and with freshly prepared 3,3′,5,5′-tetramethylbenzidine (TMB) substrate solution (0.05 mg/mL) for 30 min at RT when the reaction was stopped with 50 μL of stopping solution (2 M sulfuric acid). Absorbance was read at 450 nm, with the reference filter set to 620 nm. Before each step, the plate was washed 5 times with washing buffer. Plasma concentrations of 4-HNE protein conjugates were interpolated from the standard curve and expressed as pmol 4-HNE protein conjugates/mg of proteins.

### 2.5. Statistical Analysis

The results are expressed as medians and 25th (Q1) and 75th (Q3) percentiles and were evaluated with GraphPad Prism version 4.00 (GraphPad Software, San Diego, CA, USA). The normality of the distribution was assessed with the Kolmogorov-Smirnov test. Since most of the analyzed parameters were not normally distributed, non-parametric analyses were used. Multiple linear regression analysis was performed to examine the possible effects of age, body mass index (BMI), diagnosis, fasting glucose, cholesterol, HDL, LDL, TG, aspartate aminotransferase (AST), alanine aminotransferase (ALT), and gamma-glutamyl transferase (GGT) levels on plasma 4-HNE concentration, using 4-HNE plasma concentration as a dependent variable and other analyzed parameters as independent variables. Differences in the distribution of 4-HNE, anthropometric, and biochemical characteristics between veterans with PTSD and control subjects were evaluated using Mann-Whitney tests, while the correlations were analyzed with Spearman’s rank tests. All tests were two-tailed, and α was set at 0.05.

G*Power 3 Software was used to determine a priori sample size and actual power. For the Mann-Whitney U test (with α = 0.05; power (1 − β) = 0.80; a medium effect size ω = 0.50), the total desired sample size was 53 per group, and the actual sample size was 58 in healthy controls group and 62 for the group with diagnosed PTSD. Therefore, we had the appropriate sample size and statistical power to detect significant differences in the studied groups.

## 3. Results

Anthropometric and biochemical characteristics of healthy control subjects and patients with PTSD are presented in Table 1. There were no differences in age (*p* = 0.684) and BMI (*p* = 0.233) between the two groups of subjects. Additionally, levels of cholesterol (*p* = 0.472), HDL (*p* = 0.417), LDL (*p* = 0.975), TG (*p* = 0.732), AST (*p* = 0.573), ALT (*p* = 0.908), and GGT (*p* = 0.227) were similar between healthy individuals and PTSD subjects. However, PTSD subjects had higher levels of fasting glucose (*p* = 0.032) when compared to healthy controls (Table 1).

**Figure 1:**
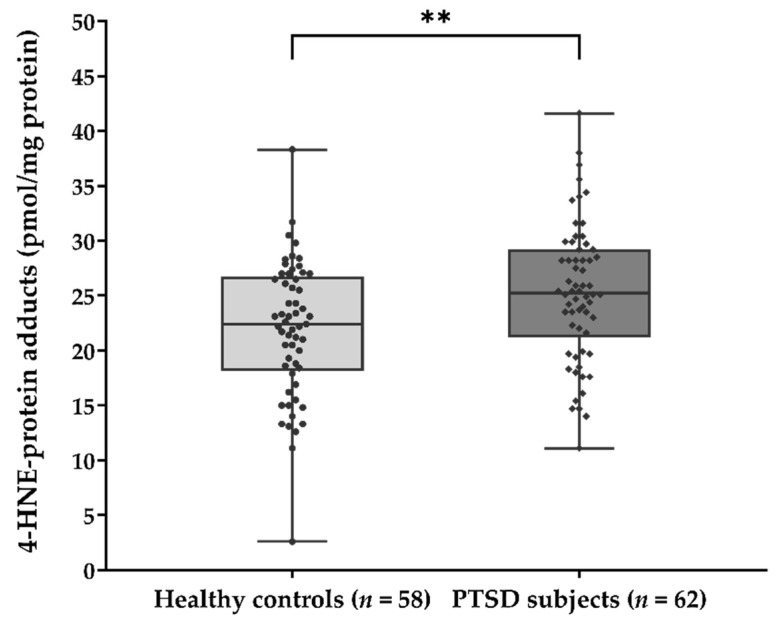
Plasma concentration of 4-HNE-protein adducts (pmol/mg protein) in samples of healthy controls (*n* = 58) and PTSD subjects (*n* = 62). Results are presented as a box and whisker plot. The median is represented by the line in the box, while the interquartile range (IQR) box represents the middle quartiles (the 75th minus the 25th percentile). The whiskers on either side of the IQR box represent the lowest and highest quartiles of the data. The ends of the whiskers represent the maximum and minimum of the data. ** *p* = 0.006. 4-HNE, 4-hydroxynonenal; PTSD, posttraumatic stress disorder.

The significant association of plasma 4-HNE concentration with the diagnosis of PTSD was confirmed by comparing the 4-HNE levels between two groups of subjects (Figure 1) using the Mann-Whitney U test (U = 1252.0; *p* = 0.006). Results suggested significantly higher concentration of HNE-protein adducts in PTSD subjects (median = 25.26 pmol/mg protein) when compared to healthy controls (median = 22.43 pmol/mg protein).

The association between the concentration of 4-HNE-protein adducts in plasma and different anthropometric and biochemical parameters was analyzed by Spearman’s rank correlation in both groups of subjects (Table 2). There was no significant correlation (Table 2) between plasma 4-HNE levels and all analyzed biochemical parameters (fasting glucose, cholesterol, HDL, LDL, TG, AST, ALT, GGT).

**Figure 2:**
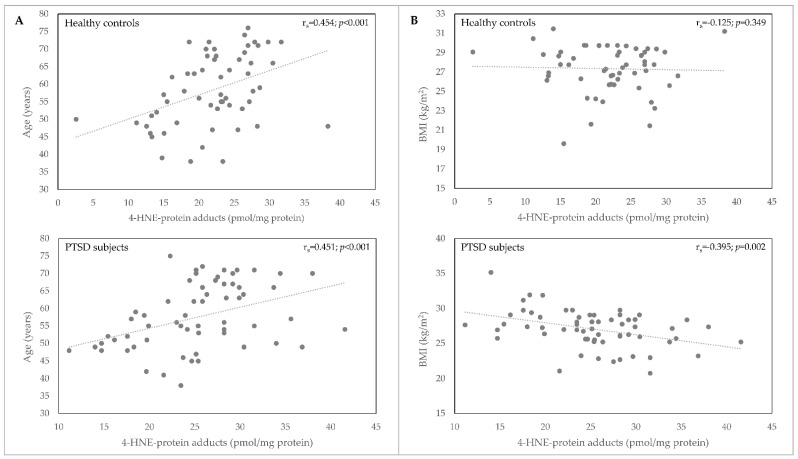
Correlation of 4-HNE-protein adducts plasma concentration (pmol/mg protein) with age (**A**) and BMI (**B**) in samples of healthy controls (*n* = 58) and PTSD subjects (*n* = 62). BMI, body mass index; 4-HNE, 4-hydroxynonenal; PTSD, posttraumatic stress disorder.

We observed a significant positive correlation between plasma 4-HNE concentration and age (*p* < 0.001) in healthy control subjects and patients with PTSD (Table 2, Figure 2). A significant negative correlation of 4-HNE plasma levels with BMI (Table 2, Figure 2) was observed only in PTSD subjects (*p* = 0.002).

To examine the possible effects of age, BMI, diagnosis of PTSD, and all biochemical parameters on plasma 4-HNE levels, multiple linear regression analysis was used. Multiple linear regression analysis, with plasma 4-HNE concentration as a dependent variable, revealed a significant model (F(11,107) = 4.25; *p* < 0.001; R_adj_^2^ = 0.232) due to significant effects of age (*p* < 0.001), BMI (*p* = 0.006), and diagnosis (*p* = 0.001) on 4-HNE levels in plasma. The model also revealed non-significant effects of fasting glucose (*p* = 0.748), cholesterol (*p* = 0.936), HDL (*p* = 0.146), LDL (*p* = 0.855), TG (*p* = 0.886), AST (*p* = 0.295), ALT (*p* = 0.338), and GGT (*p* = 0.607) concentration on plasma 4-HNE levels.

## 4. Discussion

This study examined the association of 4-HNE concentration as a measure of lipid peroxidation with the development of PTSD by comparing the concentration of 4-HNE-protein adducts in plasma samples of PTSD subjects in relation to healthy controls. Our results revealed significantly elevated levels of 4-HNE in patients with PTSD compared to healthy control subjects and suggested that the accumulation of plasma 4-HNE increases with aging. The results also indicate that plasma 4-HNE levels negatively correlate with BMI but only in individuals diagnosed with PTSD, thus confirming previous findings on the altered lipid metabolism in PTSD since such a trend was not seen in healthy control subjects [10].

It should be mentioned here that accumulation of 4-HNE in blood vessels progresses by aging, reaching its plateau between the age of 60 to 65 years [31]. Accumulation of 4-HNE in arteries, notably in the aorta, is also known to be influenced by fat-rich food and (oxidative) stress [32]. However, the accumulation of 4-HNE in the blood vessels is not necessarily irreversible, indicating that such 4-HNE-protein adducts might not only contribute to pathogenesis of atherosclerosis but could also be the source of systemic vascular stress [31,32]. Furthermore, in obese, diabetic patients, the blood-originating 4-HNE accumulates in adipose tissues and alters growth and metabolism of the (pro)adipocytes [33,34], which might cause inflammation relevant for metabolic syndrome and systemic vascular stress that is not only chronic but also acute. In favor of this assumption are recent findings revealing vascular stress as a possible cause of abundant blood-originating 4-HNE accumulation in the lungs of patients with SARS-CoV-2 infection associated with the lethal outcome of COVID-19 [35].

Studies examining the direct effect of exposure to a traumatic experience or traumatic events on oxidative stress measures in humans are scarce. However, there are indications that psychological stress in childhood and lifetime chronic stress promotes oxidative stress [36,37]. Different blood biomarkers of oxidative stress were found to be elevated in chronically stressed caregivers [38], in college students during examination periods [39,40], and in individuals who lost a spouse or a close relative [41]. Many psychiatric disorders are associated with oxidative stress [42], such as depression with increased oxidative DNA damage [43,44,45] or 4-HNE [46]. Indeed, increased levels of 4-HNE found in coronary artery disease patients with depression and their attenuation after cardiac rehabilitation associated with the improvement of depression symptoms imply its importance in the progression of depression and as a biomarker of depression symptoms [46]. Studies in patients with bipolar disorder and schizophrenia show either significantly higher [47,48] or no difference [49] in 4-HNE levels.

The involvement of oxidative stress with the diagnosis of PTSD was studied using various indicators. Decreased levels of erythrocyte SOD and GPx suggest a poorer response to oxidative stress in patients with PTSD, which could be a consequence of impaired activity and/or synthesis of these two enzymes [11]. Lipid peroxidation byproduct MDA was found to be increased in both the military population with PTSD [50] and civilian earthquake survivors who developed PTSD [12]. Both studies [12,50] suggested higher rates of lipid peroxidation and decreased antioxidant capacity associated with PTSD. Another study did not detect any significant differences in SOD, GPx, and catalase activity between patients with PTSD and healthy subjects; however, they reported a significant positive correlation between SOD activity, GPx activity, and the symptom severity in PTSD patients [51]. Tezcan and colleagues also reported a positive correlation trend between MDA levels and symptom severity [51]. A preliminary study by Michels and colleagues suggested higher levels of γ-aminobutyric acid and glutathione in the dorsolateral prefrontal cortex and anterior cingulate cortex of PTSD subjects [52]. The study that included active soldiers who took part in the Croatian war in the period between 1991 and 1994 found no relationship between oxidative damage markers and the diagnosis of chronic PTSD [15]. No correlation between oxidative stress levels and PTSD was confirmed in individuals who developed PTSD following a sexual trauma, although the results suggested a decrease in both cortisol and 8-hydroxy-2′-deoxyguanosine (8-OHdG) levels with the time that has elapsed since the trauma exposure [53].

The link between PTSD and oxidative stress is still not understood. However, the available evidence emphasizes the importance of chronic and repeated activation and dysregulation of the HPA [54] axis, which is a consequence of re-experiencing the trauma. Abnormal functioning of the HPA axis has long been associated with PTSD pathophysiology and such repeated activation of the HPA system is considered the main cause of stress-related damage to the brain [54]. The model of glucocorticoid-hippocampal atrophy [55] suggests that glucocorticoids, which are released after exposure to stress, execute their neurotoxic effects on specific brain regions, with the hippocampus being extremely sensitive to such action due to the high density of glucocorticoid receptors. The association of increased glucocorticoid levels and oxidative stress was supported by animal studies showing that the administration of glucocorticoids can affect the parameters of oxidative stress [56]. A study by Sato and colleagues showed a significant association between increased hippocampal lipid oxidation, reduced antioxidant enzyme activity, and subcutaneous corticosterone administration in rats [57]. Thus, the evidence from animal studies leads to conclusion that chronic trauma-related HPA-axis activation is a key mechanism of glucocorticoid-related oxidative stress damage and emphasizes the importance of this process in the development of PTSD. Regarding the HPA axis, glucocorticoids affect the expression of proinflammatory cytokines [58,59]. Interaction between PTSD and oxidative stress could also be mediated by the sleep disturbances, very common symptoms of PTSD. Normal sleep is necessary for restoring oxidant/antioxidant balance and ensuring metabolic homeostasis in the brain [60]. The link between sleep deprivation and oxidative stress was confirmed in the case of insomnia, demonstrating a significant increase in MDA levels and a reduction of GPx activity in subjects with insomnia compared to controls [61]. Animal studies have further contributed to elucidating the link between oxidative stress and sleep deprivation. They have suggested that the lack of sleep can lead to hippocampal oxidative stress and cause memory deficits in mice; the effect can be blocked by antioxidant agents, such as melatonin, N-tert-butyl-alpha-phenylnitrone, or vitamin E [62]. Sleep deprivation was also found to increase the expression of different proinflammatory molecules, such as C-reactive protein, tumor necrosis factor-α, and interleukins [63,64]. All these studies suggest that oxidative stress and inflammation could be the main mechanisms contributing to accelerated aging, cognitive decline, and neurodegeneration as consequences of chronic PTSD.

Our study points to the same direction with a limitation of the study involving only male subjects since the PTSD group included only male combat-exposed war veterans, while 4-HNE levels were determined only at one-time point (yet almost 30 years after the traumatic events of the war). Therefore, in further studies, civilian trauma victims and female subjects should be included. Although this study had strict exclusion criteria, one of the limitations is that we did not take into account the possibility of imbalanced nutrition state. On the other hand, the strengths of the study are in the evaluation of various factors that might affect 4-HNE levels, such as fasting glucose, AST, ALT, GGT, cholesterol, HDL, LDL, and TG. To the best of our knowledge, this is the first study that evaluated 4-HNE-protein levels in PTSD. While including subjects in the study, different comorbidities were taken into account since they often accompany the diagnosis of PTSD and are known to contribute to the elevation of general somatic oxidation state. The subjects with chronic drug abuse, alcohol dependence, diagnosis of different psychiatric disorders, and/or the use of lipid lowering agents and antihypertensive and antidiabetic medication were excluded. We also excluded potential somatic diseases, such as fibrosis, sclerosis, cirrhosis, and malignant liver disease. The included subjects comprised ethnically homogenous male populations matched and controlled for biochemical measures that might affect 4-HNE levels.

## 5. Conclusions

Our results revealed significantly elevated levels of 4-HNE in patients with PTSD. The accumulation of plasma 4-HNE seems to increase both in healthy people and in PTSD patients with aging but in a negative correlation with BMI, showing specific pattern of change for individuals diagnosed with PTSD. These findings suggest that oxidative stress and altered lipid metabolism reflected by increase of 4-HNE might be associated with PTSD.

In conclusion, since 4-HNE is already considered as important clinical biomarker of numerous stress- and age-associated diseases [65,66,67], we believe that further studies will prove its importance as a pathophysiological factor and biomarker for PTSD.

## Figures and Tables

**Table 1:** Anthropometric and biochemical characteristics of healthy controls and patients with PTSD. Data were analyzed with Mann-Whitney U test and shown as median and 25th (Q1) and 75th (Q3) percentiles.

Anthropometric and Biochemical Characteristics	Healthy Controls (*n* = 58)			PTSD Subjects (*n* = 62)			Test Statistics	
	Median	Percentiles		Median	Percentiles		U	*p*
		25th	75th		25th	75th		
Age (years)	57	49	67	56	50	66	1875.5	0.684
BMI (kg/m^2^)	27.8	26.2	29.1	27.3	25.6	28.7	2025.0	0.233
Fasting glucose (mmol/L)	4.9	4.0	5.5	5.4	4.4	5.6	1389.5	0.032
Cholesterol (mmol/L)	5.2	4.5	6.0	5.2	4.5	5.7	1934.5	0.472
HDL (mmol/L)	1.2	1.0	1.3	1.2	1.0	1.5	1645.0	0.417
LDL (mmol/L)	3.0	2.4	3.7	3.0	2.4	3.7	1792.0	0.975
TG (mmol/L)	1.6	1.1	2.4	1.6	1.3	2.3	1733.0	0.732
AST (U/L)	18.0	10.0	22.0	17.5	12.0	22.0	1691.0	0.573
ALT (U/L)	20.0	13.5	23.0	17.0	13.5	23.3	1820.0	0.908
GGT (U/L)	20.0	13.5	24.5	22.0	16.0	23.5	1542.5	0.227

AST, aspartate aminotransferase; ALT, alanine aminotransferase; BMI, body mass index; GGT, gamma-glutamyl transferase; HDL, high-density lipoprotein; LDL, low-density lipoprotein; PTSD, posttraumatic stress disorder; TG, triglyceride.

**Table 2:** Correlation of 4-HNE-protein adducts concentration (pmol/mg protein) with different anthropometric and biochemical characteristics of healthy controls and patients with PTSD. Spearman’s rank correlation was used to test the potential significant relationship between different parameters.

Anthropometric and Biochemical Characteristics	Spearman’s Rank Correlation	4-HNE-Protein Adducts (pmol/mg Protein)	
		Healthy Controls (*n* = 58)	PTSD Subjects (*n* = 62)
Age (years)	r_s_	0.454	0.451
	*p*	**<0.001**	**<0.001**
BMI (kg/m^2^)	r_s_	−0.125	−0.395
	*p*	0.349	**0.002**
Fasting glucose (mmol/L)	r_s_	−0.166	−0.157
	*p*	0.212	0.222
Cholesterol (mmol/L)	r_s_	−0.071	0.087
	*p*	0.597	0.503
HDL (mmol/L)	r_s_	0–061	−0.150
	*p*	0.635	0.243
LDL (mmol/L)	r_s_	−0.018	0.165
	*p*	0.895	0.201
TG (mmol/L)	r_s_	−0.095	0.071
	*p*	0.478	0.582
AST (U/L)	r_s_	0.050	−0.041
	*p*	0.712	0.750
ALT (U/L)	r_s_	−0.014	−0.065
	*p*	0.917	0.614
GGT (U/L)	r_s_	−0.132	0.002
	*p*	0.322	0.987

AST, aspartate aminotransferase; ALT, alanine aminotransferase; BMI, body mass index; GGT, gamma-glutamyl transferase; HDL, high-density lipoprotein; 4-HNE, 4-hydroxynonenal; LDL, low-density lipoprotein; PTSD, posttraumatic stress disorder; r_s_, Spearman’s rank correlation coefficient; TG, triglyceride. Statistically significant results (*p* ≤ 0.05) are in bold.

## Data Availability

The data presented in the study are available on request from the corresponding author (N.Ž.) and N.P. The data are not publicly available due to privacy/ethical restrictions, since they contain information that could compromise the privacy of research participants.
